# Absolute Cardiovascular Disease Risk Is Associated With the Incidence of Non-amnestic Cognitive Impairment in Japanese Older Adults

**DOI:** 10.3389/fnagi.2021.685683

**Published:** 2021-06-14

**Authors:** Keitaro Makino, Sangyoon Lee, Seongryu Bae, Ippei Chiba, Kenji Harada, Osamu Katayama, Yohei Shinkai, Hiroyuki Shimada

**Affiliations:** ^1^Department of Preventive Gerontology, Center for Gerontology and Social Science, National Center for Geriatrics and Gerontology, Obu, Japan; ^2^Japan Society for the Promotion of Science, Chiyoda-ku, Japan; ^3^Center for Gerontology and Social Science, National Center for Geriatrics and Gerontology, Obu, Japan

**Keywords:** cardiovascular disease, risk score, cognitive impairment, non-amnestic subtype, community setting

## Abstract

**Background:**

The estimated absolute cardiovascular disease (CVD) risk level is known to be a useful surrogate marker for future cognitive impairment; however, evidence regarding its predictive validity in terms of cognitive subtypes is limited. We aimed to examine subtype-dependent differences in the associations between absolute CVD risk and the incidence of cognitive impairment in a community-dwelling older Japanese cohort.

**Methods and Results:**

This study comprised 1,641 cognitively intact older Japanese participants without CVDs at baseline. We estimated absolute CVD risk using WHO region-specific risk estimation charts and included age, sex, diabetes mellitus, smoking, systolic blood pressure, and total cholesterol at baseline, and the CVD risk level was stratified into the three following risk categories: low (<10%), moderate (10 to <20%), and high (≥20%). Objective cognitive screening was performed using a multicomponent neurocognitive test at baseline and follow-up, and the incidence of cognitive impairment over 48 ± 2 months was determined. The incidence of cognitive impairment in low-, moderate-, and high-CVD risk participants was 1.2, 3.0, and 5.4%, respectively, for amnestic subtypes and 5.8, 10.1, and 14.0%, respectively, for non-amnestic subtypes. After adjusting for potential confounding factors, the absolute CVD risk level was significantly associated with non-amnestic impairment but not with amnestic impairment.

**Conclusions:**

The absolute CVD risk estimated using region-specific risk estimation charts in old age is useful to predict incidence of cognitive impairment. Strategies to screen populations at risk of cognitive impairment and to prevent progression to dementia should be cognitive subtype-specific.

## Introduction

Prevalence rates concerning Alzheimer’s disease (AD) and dementia are increasing rapidly along with an aging global population. According to predictions, the total number of people with dementia is likely to reach 82 million in 2,030 and 152 million in 2,050 (World Health Organization (WHO), 2019). Therefore, modifiable risk factors associated with dementia need to be urgently identified. Furthermore, along with efforts to determine risk factors for dementia, there is increasing interest in studying predictors of cognitive decline as it is now widely accepted that dementia has a long preclinical phase (Kaffashian et al., 2013).

Cardiovascular disease (CVD), as a modifiable risk factor for cognitive impairment or dementia, has become an area of interest. Previous studies have shown that traditional CVD risk factors including obesity, diabetes mellitus, smoking, hypertension, and hyperlipidemia are individually associated with cognitive decline (Carmelli et al., 1998). Regarding potential mechanisms, exposure to CVD risk factors might accelerate cognitive decline due to cerebral hypoperfusion, hypoxia, emboli, or infarcts, which lead to vascular and degenerative brain lesions (Qiu and Fratiglioni, 2015; Cohen, 2016). The point here is that CVD risk factors are correlated with each other, making it difficult to isolate their individual effects on cognitive decline remains challenging (Song et al., 2020).

Recently, multivariable CVD risk assessments have been advocated to estimate absolute CVD risk levels and to guide treatment concerning potential risk factors. Over the past decades, several CVD risk estimation tools involving multivariable risk factors have been developed to establish accurate estimation models for an individual’s absolute risk of a CVD event (Conroy et al., 2003; Hippisley-Cox et al., 2007; D’Agostino et al., 2008). Previous large-scale cohort studies have shown that some CVD risk estimation tools have been useful in predicting not only a CVD event but also cognitive decline (Samieri et al., 2018; Song et al., 2020) and dementia (both all-cause dementia and AD) (Viticchi et al., 2017; Fayosse et al., 2020). Therefore, absolute CVD risk, estimated using multivariable risk factors, may be a useful surrogate marker of cognitive decline.

However, most CVD risk estimation tools have been developed based on data from Western countries (Conroy et al., 2003; Hippisley-Cox et al., 2007; D’Agostino et al., 2008), and it is unclear whether these tools are applicable to Asian populations. Previous studies have reported interethnic heterogeneity in terms of CVD risks and CVD events between Asian and Western countries. For example, Asian people have been found to have a higher predisposition to insulin resistance at a lesser degree of obesity than European people (Yoon et al., 2006), and the prevalence of adult obesity in most Asian countries is relatively low compared with Western countries such as the United States (Yoon et al., 2006). Interethnic heterogeneity is considered to be affected by lifestyle, environmental factors, and genetic predisposition (Yoon et al., 2006); thus, validation of a risk estimation tool in an Asian cohort is necessary for accurate CVD risk estimation in an Asian population.

The cognitive domain has previously been divided into amnestic and non-amnestic subtypes, with amnestic impairment hypothesized as more likely to progress to dementia due to AD (Jicha et al., 2006) and non-amnestic impairment more likely to progress to vascular and other forms of non-AD dementia (Luchsinger et al., 2009). Therefore, the mechanisms underlying CVD risk and cognitive impairment appear to differ between amnestic and non-amnestic subtypes and the strategies to prevent progression to dementia should be subtype-specific. However, the difference in the association between absolute CVD risk and cognitive impairment in amnestic and non-amnestic subtypes remains unclear.

Therefore, we examined the prospective associations of absolute CVD risk, based on region-specific risk estimation charts, with the incidence of cognitive impairment in amnestic and non-amnestic subtypes among Japanese older adults without CVDs, in a 4-year longitudinal cohort study. We hypothesized that the association between absolute CVD risk and cognitive impairment would be more robust in the non-amnestic subtype than in the amnestic subtype, because CVDs could directly lead to vascular dementia.

## Materials and Methods

### Study Settings and Participants

**FIGURE 1:**
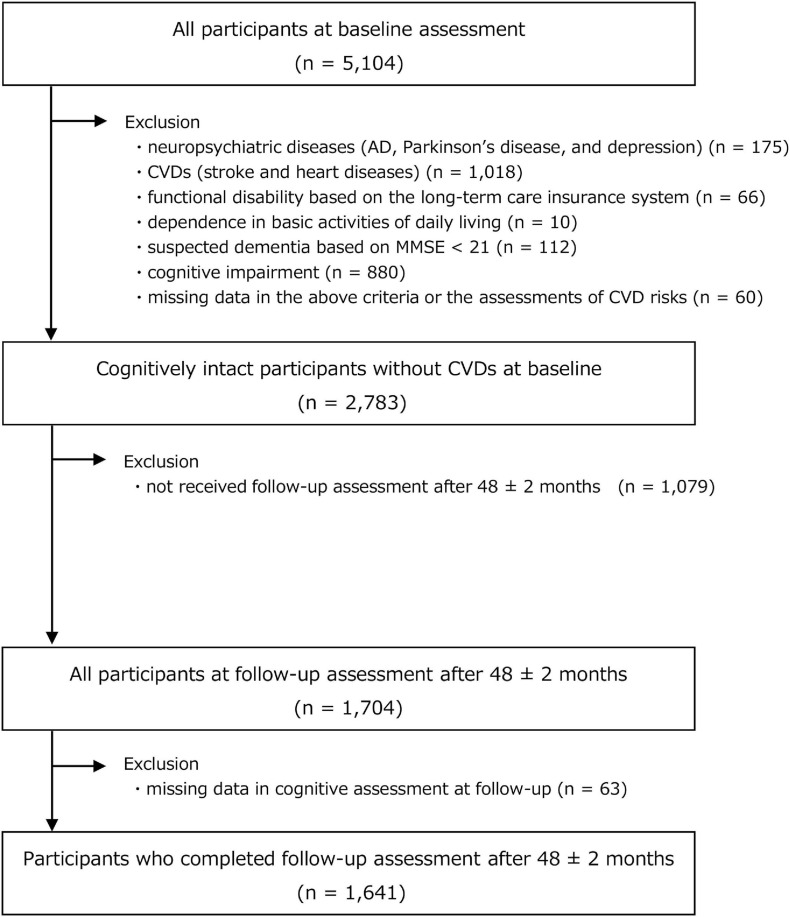
Flowchart of participant recruitment and screening.

This prospective cohort study involved community-dwelling older Japanese adults who were enrolled from a sub-cohort of the National Center for Geriatrics and Gerontology-Study of Geriatric Syndromes (NCGG-SGS). The NCGG-SGS is a Japanese national cohort study, the primary aim of which is to establish a screening system for geriatric syndromes and to validate evidence-based interventions to prevent such syndromes. Our study inclusion criteria comprised older adults (age, ≥65 years) at the time of the baseline assessment (from August 2011 to February 2012) who resided in Obu City (population of approximately 88,000), Aichi prefecture, Japan. At the registration of the Obu study cohort, individuals aged 65 years or older, living in Obu City, not hospitalized, not in residential care, not certified by the national long-term care insurance system as having a functional disability, and not participating in another study (*n* = 14,313) were sent an invitation letter. Overall, 5,104 individuals aged ≥65 years completed our baseline assessment. At baseline, our exclusion criteria comprised those with: (i) a history of neuropsychiatric diseases including AD, Parkinson’s disease, and depression (*n* = 175); (ii) a history of CVDs, including stroke and heart diseases (i.e., angina, myocardial infraction, and aortic aneurysm) (*n* = 1,018); (iii) functional disability, based on the long-term care insurance system (*n* = 66); (iv) dependence in basic activities of daily living (*n* = 10); (v) suspected dementia, based on a Mini-Mental State Examination score <21 (Excellence NIfHaC, 2011) at baseline (*n* = 112); (vi) cognitive impairment (refer to details concerning the definition, as discussed later) at baseline (*n* = 880), and; (vii) missing data in the above criteria or missing data concerning assessments for CVD risks (*n* = 60). After exclusion, we included 2,783 cognitively intact participants at baseline. Of these, 1,704 participants (61.2%) completed a follow-up assessment (from August 2015 to February 2016) that was conducted 48 ± 2 months from baseline. At this follow-up, we excluded participants with missing data for cognitive assessment (*n* = 63). Finally, data concerning 1,641 participants were available for analysis; a flow chart depicting the exclusion process is shown in Figure 1.

All baseline assessments were undertaken as health check-ups by well-trained nurses and study assistants in community centers. All staff received training from the authors in terms of the protocols for administering the assessments prior to study commencement.

### Ethical Approval

The study protocol was developed in accordance with the Helsinki Declaration and was approved by the Ethics Committee of the National Center for Geriatrics and Gerontology (NCGG). Prior to study participation, written informed consent was obtained from all participants.

### Estimation of Absolute CVD Risk

We estimated 10-year CVD risk using the revised World Health Organization (WHO) CVD risk estimation charts (2019) at baseline (WHO CVD Risk Chart Working WHO CVD Risk Chart Working Group, 2019). These estimation charts indicate the absolute risk of a CVD event according to an individual’s risk status, and a higher risk score indicates a greater risk-factor burden. The development group calibrated prediction models for 21 global regions, and region-specific prediction charts were available. The estimation charts provided two types of estimation models: a laboratory-based model including medical history and blood data, and a non-laboratory-based model that consisted of convenient variables for resource-limited settings. In this study, we used a laboratory-based risk estimation model which included age, sex, current history of diabetes mellitus, smoking status, systolic blood pressure, and total cholesterol for the high-income Asia-Pacific region, including Japan.

Regarding each component of the WHO risk estimation model, we assessed diabetes mellitus, smoking status, systolic blood pressure, and total cholesterol levels, along with age and sex. A current history of diabetes mellitus was assessed through face-to-face interviews by nurses. Nurses measured systolic blood pressure using an automated sphygmomanometer, with participants in a seated position. Total serum cholesterol levels (in mmol/L) were measured by enzyme method at a laboratory (Good Life Design Co., Japan). Smoking status was assessed as the presence or absence of regular smoking (current vs. former/never) by the study assistants. Finally, we calculated absolute CVD risk (%) based on the above risk status using revised WHO CVD risk estimation charts and we stratified the CVD risk into three categories, namely, low (<10%), moderate (10 ≤ 20%), and high (≥20%) risk (World Health Organization (WHO), 2007).

### Assessment of Cognitive Functions and Operational Criteria for Cognitive Impairment

Cognitive assessment was conducted using the NCGG–Functional Assessment Tool (NCGG-FAT) (Makizako et al., 2013). The detailed protocol of NCGG-FAT was described in a previous study (Makizako et al., 2013). The NCGG-FAT includes the following cognitive tests: (i) memory (word list memory-I [immediate recognition] and word list memory-II [delayed recall]); (ii) attention (a tablet-based version of the Trail Making Test [TMT]-part A); (iii) executive function (a tablet-based version of the TMT-part B); and (iv) processing speed (a tablet-based version of the Digit Symbol Substitution Test). Participants were given approximately 20 min to complete the test battery. This tool has previously been confirmed to have high test-retest reliability (intraclass correlation coefficients ranging from 0.79 to 0.94 in each test) and moderate-to-high validity (Pearson’s correlation coefficients between the NCGG-FAT score and that of widely used clinical tests, including the subtest of the AD Assessment Scale-cognitive [delayed recall, modified], the written TMT-parts A and B, and the Digit Symbol-Coding subtest of the Wechsler Adult Intelligence-III ranging from 0.55 to 0.84) among community-dwelling older adults (Makizako et al., 2013). All tests had established standardized thresholds for defining objective cognitive impairments in the corresponding tests (a score of ≥1.5 standard deviations [SD] below the age- and education-specific means, based on our own algorithm sourced from a database including >10,000 community-dwelling older adults), which were derived from a population-based cohort (Shimada et al., 2013).

Cognitive impairment was defined as a participant score below the standardized thresholds (a score of ≥1.5 SDs below the age- and education-specific means) in one or more cognitive tests in the follow-up assessment. Additionally, cognitive impairment was classified as amnestic or non-amnestic impairment (Petersen, 2004). The former indicated individuals with a memory deficit (non-memory domain including attention, executive function, and processing speed remains intact) and the latter indicated individuals with a deficit in either attention, executive function, or processing speed (memory remains intact) in our study (Petersen, 2004).

### Potential Confounding Factors

As covariates, education level, a medical history of pulmonary disease (i.e., pneumonia, tuberculosis, and chronic obstructive pulmonary disease), and the number of prescribed medications (total of overall drugs continuously prescribed by a doctor) were assessed through face-to-face interviews at baseline. We also included body mass index, alcohol consumption habits, slow gait speed, depressive symptoms, physical inactivity, living arrangements (living alone or cohabiting), employment status (the presence of paid work), and global cognitive function at baseline as covariates. Body mass index was calculated as bodyweight (kg) divided by the square of body height (m^2^). Current alcohol consumption habits were assessed as the presence or absence of regular alcohol consumption (current vs former/never). Gait speed was measured in seconds using a stopwatch. Participants were asked to walk on a flat and straight surface at a comfortable walking speed. Two markers were used to indicate the start and end of a 2.4-m walk path, with a 2-m section to be traversed before passing the start marker so that participants were walking at a comfortable pace by the time they reached the timed path. Participants were asked to continue walking for an additional 2-m distance past the end of the path to ensure a consistent walking pace while on the timed path (Shimada et al., 2013; Doi et al., 2015). A gait speed <1.0 m/s was defined as a slow gait speed (Doi et al., 2015). Depressive symptoms were assessed using the 15-item Geriatric Depression Scale (GDS). The GDS was developed specifically for the screening of depression among elderly individuals and is used to quantify depressive symptoms. Participants could respond “yes” or “no” to 15 questions; thus, the total score ranged from 0 to 15. Participants who scored ≥6 on the GDS were considered to have depressive symptoms in this study (Ezzati et al., 2019). Physical inactivity was evaluated using the following questions: (i) “Do you engage in more than moderate levels of physical exercise or sports aimed at health?” and (ii) “Do you engage in low levels of physical exercise aimed at health?” Participants who responded “no” to both questions were defined as being inactive (Shimada et al., 2013). Global cognitive function was measured using the Mini-Mental State Examination (Folstein et al., 1983); and scores ranged from 0 to 30, with higher scores indicating better cognitive performance (Folstein et al., 1983).

### Statistical Analysis

Baseline characteristics were compared between participants who completed the follow-up assessment and participants who were lost to follow-up using the Student’s *t*-test for continuous variables and a χ^2^ test for categorical variables. Furthermore, we compared baseline characteristics according to CVD risk levels (low-, moderate-, and high-risk levels) using a one-way analysis of variance for continuous variables and a χ^2^ test for categorical variables. We then examined the association between baseline CVD risk levels and the incidence of cognitive impairment after 4 years. For this analysis, we used a χ^2^ test and logistic regression analysis because we dealt with non-time series data. In this analysis, we first examined CVD risk levels and the incidence of cognitive impairment (regardless of cognitive subtype) in all participants. Second, we examined CVD risk levels and the incidence of cognitive impairment divided according to cognitive subtype (amnestic or non-amnestic impairment). In the latter analysis, we compared participants who had remained cognitively intact over 4 years as a reference group and those who showed cognitive impairment only in the amnestic subtype or only in non-amnestic subtype to clarify the subtype-dependent difference in the association between CVD risk levels and the incidence of cognitive impairment in the true sense. Logistic regression analysis was used to calculate odds ratios (ORs) and 95% confidence intervals (CIs) at each CVD risk level at baseline and in relation to the incidence of cognitive impairment after 4 years, and univariate (crude) and multivariate (adjusted) regression models were developed. The multivariate regression model was adjusted for all potential confounding factors assessed in this study.

All analyses were performed using IBM SPSS Statistics 25 (IBM Japan, Tokyo, Japan) software. The level of statistical significance was set to *P* < 0.05.

## Results

### Participants’ Baseline Characteristics

Of the 2,783 cognitively intact participants at baseline, 1,704 (61.2%) completed a follow-up assessment. Compared with participants who completed the follow-up assessment, those who were lost to follow-up assessment were significantly older (*P* < 0.001), had a significantly lower proportion of female subjects (*P* = 0.014), higher proportion of diabetes mellitus (*P* = 0.007) and current smokers (*P* = 0.013), had significantly higher systolic blood pressure (*P* < 0.001) and more prescribed medications (*P* = 0.002), had a significantly higher proportion of slow gait speed (*P* < 0.001), depressive symptoms (*P* < 0.001), and physical inactivity (*P* < 0.001), and showed a significantly lower MMSE score (*P* < 0.001, Table 1).

**TABLE 1:** Comparison of baseline characteristics between participants who completed follow-up assessment and participants who were lost to follow-up assessment.

		**Completed follow-up *n* = 1,704**	**Lost to follow-up *n* = 1,079**	***P***
**Components of WHO risk estimation model**				
Age	(Years)	70.8 ± 4.6	71.8 ± 5.6	<0.001
Female	(*n*, %)	939 (55.1)	543 (50.3)	0.014
Diabetes mellitus	(*n*, %)	153 (9.0)	131 (12.1)	0.007
Smoking status	(*n*, %)	150 (8.8)	126 (11.7)	0.013
Systolic blood pressure	(mmHg)	140.0 ± 20.2	144.6 ± 21.7	<0.001
Total cholesterol	(mmol/l)	5.5 ± 0.8	5.5 ± 0.9	0.431
**Other characteristics**				
Education level	(Years)	11.8 ± 2.5	11.6 ± 2.4	0.550
Pulmonary disease	(*n*, %)	198 (11.6)	113 (10.5)	0.349
Prescribed medication	(Number)	1.5 ± 1.6	1.7 ± 1.9	0.002
Body mass index	(kg/m^2^)	22.8 ± 2.9	23.0 ± 3.3	0.055
Drinking habit	(*n*, %)	822 (48.2)	494 (45.8)	0.206
Slow gait speed	(*n*, %)	66 (3.9)	109 (10.1)	<0.001
Depressive symptoms	(*n*, %)	162 (9.5)	154 (14.3)	<0.001
Physical inactivity	(*n*, %)	415 (24.4)	341 (31.7)	<0.001
Living alone	(*n*, %)	160 (9.4)	106 (9.8)	0.704
Employment	(*n*, %)	528 (31.0)	340 (31.5)	0.771
MMSE	(Score)	27.0 ± 2.2	26.6 ± 2.3	<0.001

*CVD, cardiovascular disease; MMSE, mini-mental state examination; WHO, World Health Organization. Data are expressed as mean ± standard deviation or numbers (%) P-values are based on the Student’s t-test for continuous variables and χ^2^ tests for categorical variables.*

Of 1,641 individuals who were included in our longitudinal analysis, the group classification according to CVD risk levels was as follows: (i) low CVD risk (*n* = 372, 22.7%), (ii) moderate CVD risk (*n* = 1,116, 68.0%), and; (iii) high CVD risk (*n* = 153, 9.3%).

The differences in baseline characteristics between the three CVD risk levels are shown in Table 2. There were significant differences in all components of the WHO risk estimation model: age (*P* < 0.001), sex (*P* < 0.001), the prevalence of diabetes mellitus (*P* < 0.001), the proportion of current smokers (*P* < 0.001), systolic blood pressure (*P* < 0.001), and total cholesterol (*P* = 0.028). In addition, there were significant differences between CVD risk levels in the number of prescribed medications (*P* < 0.001), body mass index (*P* < 0.001), the proportion of participants who consumed alcohol (*P* < 0.001), the prevalence of a slow gait speed (*P* = 0.003), physical inactivity (*P* = 0.001), and the Mini-Mental State Examination score (*P* < 0.001).

**TABLE 2:** Baseline characteristics according to estimated CVD risk levels.

		**Overall *n* = 1,641**	**CVD risk levels**			***P***
			**Low risk (<10%) *n* = 372**	**Moderate risk (10 ≤ 20%) *n* = 1,116**	**High risk (≥20%) *n* = 153**	
**Components of WHO risk estimation model**						
Age	(Years)	70.9 ± 4.7	68.0 ± 3.0	71.6 ± 4.7	72.6 ± 5.0	<0.001
Female	(*n*, %)	900 (54.8)	363 (97.6)	515 (46.1)	22 (14.4)	<0.001
Diabetes mellitus	(*n*, %)	147 (9.0)	3 (0.8)	87 (7.8)	57 (37.3)	<0.001
Smoking status	(*n*, %)	146 (8.9)	1 (0.3)	81 (7.3)	64 (41.8)	<0.001
Systolic blood pressure	(mmHg)	140.1 ± 20.1	125.8 ± 15.3	141.7 ± 18.1	162.9 ± 18.5	<0.001
Total cholesterol	(mmol/l)	5.5 ± 0.8	5.6 ± 0.8	5.4 ± 0.8	5.6 ± 0.9	0.028
**Other characteristics**						
Education level	(Years)	11.8 ± 2.5	11.8 ± 2.1	11.7 ± 2.6	11.9 ± 2.5	0.734
Pulmonary disease	(*n*, %)	193 (11.8)	34 (9.1)	138 (12.4)	21 (13.7)	0.180
Prescribed medication	(Number)	1.5 ± 1.6	1.2 ± 1.4	1.6 ± 1.7	1.7 ± 1.8	<0.001
Body mass index	(kg/m^2^)	22.7 ± 2.9	22.1 ± 2.8	22.9 ± 2.9	23.3 ± 3.0	<0.001
Drinking habit	(*n*, %)	789 (48.1)	132 (35.5)	563 (50.4)	94 (61.4)	<0.001
Slow gait speed	(*n*, %)	65 (4.0)	4 (1.1)	51 (4.6)	10 (6.5)	0.003
Depressive symptoms	(*n*, %)	158 (9.6)	33 (8.9)	111 (10.0)	14 (9.2)	0.808
Physical inactivity	(*n*, %)	396 (24.1)	69 (18.6)	277 (24.8)	50 (33.3)	0.001
Living alone	(*n*, %)	155 (9.4)	40 (10.8)	100 (9.0)	15 (9.8)	0.585
Employment	(*n*, %)	507 (30.9)	121 (32.5)	338 (30.3)	48 (31.4)	0.714
MMSE	(Score)	27.0 ± 2.2	27.5 ± 2.1	26.9 ± 2.2	26.6 ± 2.2	<0.001

*CVD, cardiovascular disease; MMSE, mini-mental state examination; WHO, World Health Organization. Data are expressed as mean ± standard deviation or numbers (%). P-values are based on one-way analysis of variance for continuous variables and χ^2^ tests for categorical variables.*

### Prospective Associations Between CVD Risk Levels and Cognitive Decline

**FIGURE 2:**
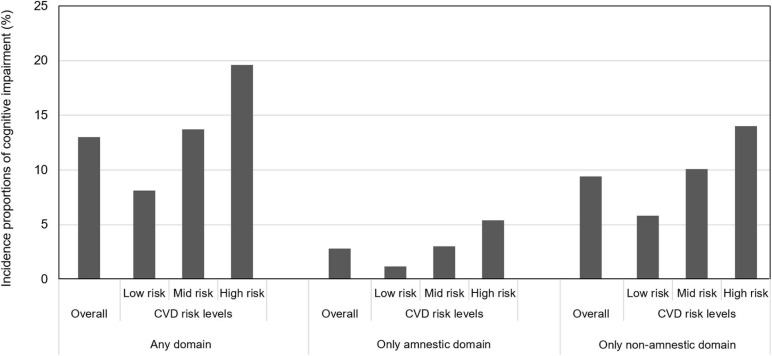
Incidence proportions of cognitive impairment according to baseline CVD risk levels.

The incidence of cognitive impairment according to baseline CVD risk levels is shown in Figure 2. Of 1,641 cognitively intact participants without CVDs at baseline, 213 (13.0%) participants had newly developed cognitive impairment in any domain after 4 years. The percentages for those with cognitive impairment in the low-, moderate-, and high-CVD risk categories were 8.1, 13.7, and 19.6%, respectively, and χ^2^ test results showed that CVD risk levels at baseline were significantly associated with the incidence of cognitive impairment (*P* = 0.001). In the cognitive subtype analysis, of 1,641 cognitively intact participants without CVDs at baseline, 41 (2.4%) participants showed cognitive impairment only in the amnestic subtype and 149 (9.1%) participants showed cognitive impairment only in the non-amnestic subtype. The percentages for those with amnestic impairment in the low-, moderate-, and high-CVD risk categories were 1.2, 3.0, and 5.4%, respectively, and the percentages for those with non-amnestic impairment in the low-, moderate-, and high-CVD risk categories were 5.8, 10.1, and 14.0%, respectively. χ^2^ test results indicated that CVD risk levels at baseline were significantly associated with the incidence of both amnestic and non-amnestic impairment (*P* = 0.033 and *P* = 0.008, respectively).

Logistic regression analysis showed that baseline CVD risk levels (reference: low risk) were significantly associated with the incidence of cognitive impairment (in any domain) after 4 years in both the crude model (moderate risk, OR 1.81; 95% CI 1.20–2.73; high risk, OR 2.78, 95% CI 1.61–4.80) and the adjusted model (moderate risk, OR 1.56, 95% CI 1.02–2.38; high risk, OR 2.21, 95% CI 1.24–3.95; Table 3). In the logistic regression analysis divided by cognitive subtype, the incidence of amnestic impairment was significantly associated with a high risk of CVD (OR 4.87, 95% CI 1.40–16.91) but not with a moderate risk of CVD (OR 2.66, 95% CI 0.93–7.62) in the crude model, and there was no significant relationship between CVD risk levels and the incidence of amnestic impairment in the adjusted model (moderate risk, OR 1.88, 95% CI 0.64–5.48; high risk, OR 3.21, 95% CI 0.89–11.57). However, incidence of non-amnestic impairment was significantly associated with moderate and high risk in both the crude model (moderate risk, OR 1.83, 95% CI 1.13–2.96; high risk, OR 2.65, 95% CI 1.39–5.05) and in the adjusted model (moderate risk, OR 1.66, 95% CI 1.01–2.73; high risk, OR 2.40, 95% CI 1.22–4.73; Table 4).

**TABLE 3:** Odds ratios and 95% confidence intervals for incidence of cognitive impairment according to CVD risk levels.

		**Cognitive impairment**			
		**Crude model**		**Adjusted model**	
		**OR**	**95% CI**	**OR**	**95% CI**
**Cardiovascular disease risk**					
Low risk (<10%)		ref		ref	
Moderate risk (10 ≤ 20%)		1.81	1.20–2.73	1.56	1.02–2.38
High risk (≥20%)		2.78	1.61–4.80	2.21	1.24–3.95
**Potential confounding factors**					
Education level	(Years)			0.94	0.88–1.00
Pulmonary disease	(*n*, %)			0.82	0.51–1.31
Prescribed medication	(Number)			1.10	1.01–1.20
Body mass index	(kg/m^2^)			0.96	0.91–1.01
Alcohol consumption habit	(*n*, %)			1.21	0.89–1.65
Slow gait speed	(*n*, %)			0.77	0.35–1.68
Depressive symptoms	(*n*, %)			1.18	0.74–1.90
Physical inactivity	(*n*, %)			1.09	0.77–1.53
Living alone	(*n*, %)			1.36	0.85–2.19
Employment	(*n*, %)			0.73	0.52–1.03
MMSE	(Score)			0.84	0.79–0.90

*CI, confidence interval; CVD, cardiovascular disease; MMSE, mini-mental state examination; OR, odds ratio. The adjusted model was adjusted for all potential confounding factors assessed in the present study.*

**TABLE 4:** Odds ratios and 95% confidence intervals for incidence of cognitive impairment in each subtype according to CVD risk levels.

		**Amnestic impairment**				**Non-amnestic impairment**			
		**Crude model**		**Adjusted model**		**Crude model**		**Adjusted model**	
		**OR**	**95% CI**	**OR**	**95% CI**	**OR**	**95% CI**	**OR**	**95% CI**
**Cardiovascular disease risk**									
Low risk (<10%)		Ref		Ref		Ref		Ref	
Moderate risk (10 ≤ 20%)		2.66	0.93–7.62	1.88	0.64–5.48	1.83	1.13–2.96	1.66	1.01–2.73
High risk (≥20%)		4.87	1.40–16.91	3.21	0.89–11.57	2.65	1.39–5.05	2.40	1.22–4.73
**Potential confounding factors**									
Education level	(Years)			1.00	0.88–1.14			0.91	0.85–0.98
Pulmonary disease	(*n*, %)			1.88	0.86–4.10			0.55	0.29–1.02
Prescribed medication	(Number)			1.02	0.83–1.24			1.16	1.05–1.27
Body mass index	(kg/m^2^)			1.05	0.94–1.17			0.93	0.87–0.99
Alcohol consumption habit	(*n*, %)			1.75	0.90–3.42			1.13	0.79–1.61
Slow gait speed	(*n*, %)			0.45	0.06–3.47			0.70	0.27–1.83
Depressive symptoms	(*n*, %)			2.03	0.85–4.86			1.12	0.64–1.95
Physical inactivity	(*n*, %)			0.97	0.47–2.03			1.06	0.71–1.58
Living alone	(*n*, %)			1.18	0.40–3.48			1.16	0.66–2.05
Employment	(*n*, %)			0.85	0.41–1.75			0.71	0.47–1.06
MMSE	(Score)			0.76	0.66–0.87			0.90	0.83–0.97

*CI, confidence interval; CVD, cardiovascular disease; MMSE, mini-mental state examination; OR, odds ratio. The adjusted model was adjusted for all potential confounding factors assessed in the present study.*

## Discussion

Our longitudinal analysis indicated that absolute CVD risk, based on WHO risk estimation charts in old age, was significantly associated with the incidence of cognitive impairment among older adults without CVDs at baseline. Additionally, after adjusting for potential confounding factors, absolute CVD risk was found to be significantly associated with non-amnestic impairment but not with amnestic impairment. These results suggested that the association between absolute CVD risk and cognitive decline differed between amnestic and non-amnestic subtypes in older Japanese.

Previous studies have shown that CVD risk estimation based on multivariable risk assessment is a useful tool to predict not only a CVD event but also cognitive decline (Samieri et al., 2018; Song et al., 2020). Samieri et al. (2018) showed that baseline cardiovascular health levels, estimated according to the American Heart Association’s Life’s Simple seven metrics (smoking, body mass index, physical activity, diet, total cholesterol, fasting glucose, and blood pressure), predicted rates of cognitive impairment. Song et al. (2020). showed that the Framingham General Cardiovascular Risk Score (FGCRS), consisting of age, sex, smoking, blood pressure, medication for hypertension, total cholesterol, high-density lipoprotein cholesterol, and diabetes mellitus, predicted rates of cognitive impairment. Our results accorded with findings from these earlier studies. Importantly, components of a CVD risk estimation model are easily obtainable in clinical and research settings and may be useful for identifying individuals at the highest risk of future cognitive impairment and dementia. Moreover, to our knowledge, this study is the first to show the predictive validity of a region-specific CVD estimation model for the incidence of cognitive impairment in a non-Western country. In Japan, the average life span is approximately 81 years for men and 87 years for women (Ministry of Health, Labour and Welfare, 2019) and identifying effective interventions to expand healthy life expectancy remains an urgent issue. Further external validation of existing and international CVD risk estimation tools to predict cognitive impairment among older adults in non-Western countries is needed.

Participants in our study were followed up at 48 ± 2 months, whereas earlier studies involved relatively longer follow-up times [i.e., the maximum follow-up period was 16.6 years in Samieri et al.’s (2018) study and 21 years in Song et al.’s (2020) study]. Given more extended average life spans in many countries, early screening and intervention to mitigate the adverse effects of prolonged exposure to CVD risks in old age is essential, although control of CVD risk beginning early in life is optimal. Therefore, a relatively short-term CVD risk estimation in old age may be becoming increasingly important along with a longer-term estimation in middle age. Our results may have clinical significance because our study findings showed the potential for absolute CVD risk to predict relatively short-term cognitive decline in old age.

Most significantly, our study findings showed a possibility that associations between absolute CVD risk levels and cognitive impairment differ between amnestic and non-amnestic subtypes. While univariable analysis showed significant associations between CVD risk level and both amnestic and non-amnestic impairment, our multivariable analysis showed that the CVD risk level was significantly associated with non-amnestic impairment but not with amnestic impairment. Some previous longitudinal studies have examined the association between CVD risk level, calculated using multivariable risk factors, and cognitive impairment in amnestic and non-amnestic subtypes, and have reported contrasting results. Aljondi et al. (2020) reported that a high FGCRS-based CVD risk level was associated with executive function but not with episodic memory, semantic memory, and visuospatial ability. Kaffashian et al. (2013) showed that a high FGCRS-based CVD risk level was associated with faster cognitive decline in reasoning, phonemic fluency, semantic fluency, and vocabulary, but not with memory. Song et al. (2020) reported that a high FGCRS-based CVD risk level was associated with cognitive decline in episodic memory and working memory, as well as visuospatial ability and perceptual speed. However, regarding individual CVD risk factors, a subtype-dependent difference in the association between individual CVD risk factors and cognitive impairment has been observed. Reitz et al. found that hypertension predicted all-cause mild cognitive impairment (MCI) and non-amnestic MCI but not amnestic MCI (Reitz et al., 2007). Bae et al. (2017) reported that metabolic syndrome was associated with non-amnestic MCI but not with amnestic MCI. Our results concerning subtype-dependent differences in the association between CVD risk and cognitive impairment appear consistent with these previous findings.

The mechanisms involved in the subtype-dependent association between CVD risk and cognitive impairment are not fully understood; however, an earlier study suggested that the risk factors for vascular dementia characterized by non-amnestic impairment were almost identical to risk factors for CVD (O’Brien et al., 2003). Common risk factors, such as cortical or subcortical infarcts and small-vessel disease (O’Brien et al., 2003), might help explain the robust association found between CVD risk level and cognitive impairment in the non-amnestic subtype in contrast to the amnestic subtype in our study. However, AD and vascular cognitive impairment are known to share common pathology, including atherosclerosis and amyloid angiopathy, and clear discrimination is difficult. Additional studies including biomarkers related to dementia subtypes, such as amyloid status and brain magnetic resonance imaging, are required. As another explanation, one previous study reported that cognitive impairment in terms of episodic memory and working memory were linked with hippocampal volume; that is, typical markers of AD-related neurodegeneration and cognitive impairment in perceptual speed are linked with white matter hyperintensities that indicate microvascular lesions in cerebral white matter (Song et al., 2020). Furthermore, Wardlaw et al. (2013) noted the possibility that the effects of CVD risk factors on brain structure begin with white matter lesions of presumed vascular origin and then proceed to morphological neurodegenerative changes. Our results, according to cognitive subtype, might reflect temporal and regional differences in terms of adverse effects of CVD risk factors on brain function.

Absolute CVD risk estimated using region-specific CVD risk prediction charts in old age was found to be useful to predict the incidence of cognitive impairment, and the association between CVD risk levels and cognitive impairment was more significant in the non-amnestic subtype. Subtype-dependent differences in the association between absolute CVD risk and cognitive impairment in the present study may provide useful information for planning tailor-made strategies to prevent dementia.

### Strengths and Limitations

A major strength of this study was that it was the first to examine the predictive validity of revised WHO CVD risk estimation charts in a non-Western country. Furthermore, we analyzed well-characterized cohort data and conducted multivariable analyses, adjusting for multiple confounding factors. Moreover, as we analyzed population-based data concerning older adults without CVDs at baseline, our findings can be generalized to community dwelling people in primary care settings.

However, our study had some limitations. First, approximately 39% of the participants dropped out during the follow-up period and their baseline characteristics were significantly different from those of participants who completed the follow-up assessment. This selection bias may have led to an underestimation of CVD risk and cognitive decline. Additionally, the number of new cases of cognitive impairment was limited particularly in subgroup analysis into amnestic and non-amnestic groups. This relatively small sample size might have affected our findings through decreased statistical power. Second, we did not undertake a comparison between the revised WHO CVD risk charts and other existing CVD risk estimation tools; therefore, we cannot confirm concurrent validity or discuss the relative merits of the WHO CVD risk charts. Third, we did not measure biomarkers related to the prognosis of MCI, such as apolipoprotein E genotype or amyloid status. Additional longitudinal studies to assess the relationships between CVD risk levels and cognitive decline in each domain using biomarkers reflecting the pathology of cognitive impairment are required. Finally, although we used the risk estimation model developed for “the high-income Asia-Pacific region” in accordance with region classification by WHO, our sample came from single country (only Japan). Therefore, there might be potential effects of socio-economic status on our findings and further examination is required to clarify whether our findings can be generalized to other countries.

## Conclusion

In conclusion, absolute CVD risk estimated according to region-specific CVD risk estimation charts in old age was useful to predict the incidence of cognitive impairment among older Japanese adults. The absolute CVD risk level can be estimated using variables that are easily obtainable in clinical and research settings, and should be used for more effective early dementia risk screening. Additionally, the association between CVD risk level and cognitive impairment was more significant in the non-amnestic subtype, and strategies to screen populations at risk of cognitive impairment and to prevent progression to dementia should be cognitive subtype-specific.

## Data Availability Statement

The datasets presented in this article are not readily available because participants of this study did not agree for their data to be shared publicly. Requests to access the datasets should be directed to KM, kmakino@ncgg.go.jp.

## Ethics Statement

The studies involving human participants were reviewed and approved by the Ethics Committee of the National Center for Geriatrics and Gerontology. The patients/participants provided their written informed consent to participate in this study.

## Author Contributions

KM designed the study, analyzed and interpreted the data, and wrote and edited the manuscript. HS administered the project, acquired funding, and reviewed and edited the manuscript. SL, SB, and KH contributed to acquisition, analysis, and interpretation of data, and reviewed and edited the manuscript. IC, OK, and YS contributed to the discussion and reviewed and edited the manuscript. All authors contributed to the article and approved the submitted version.

## Conflict of Interest

The authors declare that the research was conducted in the absence of any commercial or financial relationships that could be construed as a potential conflict of interest.
